# Distinct Second-to-Fourth Digit Ratio Among Patients With Schizophrenia

**DOI:** 10.7759/cureus.83446

**Published:** 2025-05-04

**Authors:** Yulei Guo, Rong Li

**Affiliations:** 1 Institute of System Science, National University of Singapore, Singapore, SGP; 2 School of Mathematics and Physics, Xi'an Jiaotong-Liverpool University, Suzhou, CHN

**Keywords:** 2d:4d ratio, mental health, schizophrenia, sex hormones, sexual dimorphism

## Abstract

Background: It has been suggested that prenatal sex hormones may have a substantial impact on brain development and mental illness. The second-to-fourth digit (2D:4D) ratio, a critical retrospective marker for the secretion of prenatal sex hormones, can be measured and compared between healthy people and patients with schizophrenia as a direct way to support the hypothesis.

Aim: This study aimed to examine whether sexual dimorphism is held in a 2D:4D ratio and further test the hypothesis that patients with schizophrenia have distinct 2D:4D ratios from healthy people.

Setting: The study was conducted at a local hospital in a typical mid-size city in the eastern part of China.

Methods: The study was performed by analyzing the 2D:4D ratios in both hands from the control group and patient group, respectively, with the application of statistical measurements (means, variances, and correlations), the independent-sample t-test, and the paired-samples t-test. In terms of the sample size, a total of 108 healthy participants were included in our study, comprising 33 men and 75 women, with a mean age of 30.55 years. Besides, there were 126 individuals with schizophrenia in the study, comprising 73 men and 53 women, with an average age of 44.2 years.

Results: The results showed that the 2D:4D ratio of women in the control group (mean value of the left hand: 0.9650; mean value of the right hand: 0.9562) was significantly higher than that of men (mean value of the left hand: 0.9418; mean value of the right hand: 0.9298), indicating that the sexual dimorphism is held in the 2D:4D ratio (left hand p-value: 0.003<0.05; right hand p-value: 0.004<0.05). This effect, however, was not observed in the patient group. Instead, the female patients displayed more masculine digit ratios, whereas the male patients' hands displayed fewer masculine patterns.

Conclusion: The results provide evidence that the pathogenesis of schizophrenia is related to abnormal prenatal sex hormone secretion. The research on the 2D:4D ratio as a biomarker should be able to help contribute to the literature on the pathogenesis of schizophrenia.

## Introduction

Schizophrenia is a neurodevelopmental mental illness. Its symptoms include hallucinations and delusions. The symptoms and behaviors of schizophrenia can bring about severe impacts to surrounding individuals and social life, including inappropriate lack of mood during conversations, stereotyped or purposeless motor movements, repeated speech, and isolation from home and relatives [1]. Every year, between 1,000,000 and 1,500,000 people around the world are diagnosed with schizophrenia [2,3]. Researchers have discovered evidence for serious abnormalities from genetics, brain structure, and environmental factors, but the actual origin of schizophrenia is unknown [1]. Researchers have concluded that hormones were crucial points that played a role in the development, prognosis, and clinical manifestations of schizophrenia. There exist many complex interrelationships between the endocrine system and the nervous system. The prenatal hormone, gonadal hormones, prolactin, cortisol, thyroid hormones, dopamine, serotonin, and glutamate influence the whole development of psychosis [4]. Intrauterine androgen levels, for example, have been connected to the release of neurotransmitters and neuromodulators, as well as the morphological and functional architecture of the developing brain [5]. Furthermore, the physical and mental adaptations caused by the exposure to androgens during early intrauterine development stayed stably throughout life [6].

A study on the pathophysiology of mental illnesses by Castle and Murray illustrated that sexual dimorphism in brain development could result in gender differences in psychopathology. The increased disinhibition and the decreased affect regulation that are already created during the intrauterine development period could be the results [7]. The sexually dimorphic nucleus (SDN) in the preoptic area of the human hypothalamus, which plays an important role in sex identity, reflects sexual dimorphism [8]. Sexual dimorphism was also seen in cognitive performance, neuroprotection, motor skills, and coordination [9]. Crow concluded that loss of lateralization to the left hemisphere of the phonological component in language can be mirrored on the masculine characteristics in schizophrenia patients [10].

In recent years, the second-to-fourth digit (2D:4D) ratio has been proposed as a biological marker of prenatal sex hormone release. The lengths of the second (index) and fourth (ring) fingers were one of the ways to portray individuals' personalities and disease risk, according to Wang et al.'s research [11]. The 2D:4D ratio is shown to be sexually dimorphic in humans, with a smaller ratio in men than women [12]. It was discovered that the digit ratio (2D:4D) and two essential sex hormones (testosterone and estrogen) had a negative relationship with prenatal testosterone and a positive relationship with prenatal estrogen [13]. Furthermore, considerable evidence for the impact of androgen can be seen in the link between the 2D:4D ratio and congenital adrenal hyperplasia (CAH), a disorder characterized by excessive androgen secretion. Individuals with CAH had a lower 2D:4D ratio than healthy people [13]. Due to the persistent organizational effects on brain activity, the vertebrate Hox genes, which were crucial throughout baby development, could explain the link between the sex hormone and the digit ratio, notably for the axial skeleton and limb development [8]. Some evidence suggests that the digit ratio difference in the right hand was more significant than in the left hand in some studies. However, the considerable dimorphism in the right hands could not be found in a specific study comparing the digit ratio of neonates at the moment of birth with those of their mothers [13]. Geetha et al. [14] discovered that sexual dimorphism was more noticeable in the right-hand ratio than in the left-hand ratio. However, a more recent study focusing on the people of North India found no significant 2D:4D ratio disparity between the right and left hands [15].

The second finger was significantly shorter than the fourth, which is a common trend among right-handed men [9]. When the ratio is less than 1, the male pattern in one's hands will be discovered. The feminine pattern, on the other hand, indicates that the 2D:4D ratio is more than 1 [16]. According to Procopio et al.'s investigation of the 2D:4D ratio in schizophrenics for different genders, the digit ratios of women in both groups (patients and controls) were considerably higher than those of men [17]. However, the female patients did not show the usual feminine pattern. In contrast, when 80 healthy people and 80 schizophrenia patients were compared, a more feminine phenotype of second and fourth digits was seen in both male and female patients than in the control group [18]. Furthermore, according to Qian et al.'s study, the patients had a less masculine pattern, with the mean values of the 2D:4D ratio in both hands being significantly higher than the controls [19].

Some researchers have concluded that the 2D:4D ratio remained stable thereafter. According to Manning et al., the digit ratio was invariant at least before age two years [5]. Besides, in one study relevant to this study about computing the correlation between age and age of onset in the control and patient groups, they did not attain any significant correlations as well [18]. However, some studies have raised questions about whether this ratio might change with age or be influenced by postnatal factors. According to Manning and colleagues' research, the digit ratios that are influenced by sex steroid levels throughout brain organization arise early in pregnancy and remain stable throughout the fetal period and even after delivery [20,17]. Understanding this relationship is crucial, as it could provide insights into the stability of the digit ratio as a biomarker and its potential sensitivity to developmental or hormonal changes later in life. Previous studies have suggested that the 2D:4D ratio is largely invariant after the age of two, indicating that it may serve as a stable indicator of prenatal hormone exposure [5]. However, other research has reported weak correlations between age and the 2D:4D ratio, particularly in men, which may be attributed to the influence of circulating postnatal sex hormones [11]. Interestingly, some evidence also suggests that the 2D:4D ratio may increase from birth to adulthood. For example, studies comparing the digit ratios of mothers and their newborns have observed significantly larger ratios in mothers, suggesting a potential age-related increase [13]. However, these findings remain inconsistent, and the mechanisms underlying such changes, if they occur, are not well-understood.

The main goals of this study were to look into the relationships between sex, age, and measured digit ratio (2D:4D) under the influence of sex hormones (testosterone and estrogen), as well as to see if the hands of patients with schizophrenia had a distinct pattern from the healthy ones. These could be a reasonable, observable, and convenient continuing research that helps to understand the pathogenesis and predict disease vulnerability in schizophrenia.

## Materials and methods

Study design and participant recruitment

This study employed a cross-sectional design to investigate the relationship between the 2D:4D ratio and schizophrenia. A total of 234 participants were recruited, consisting of 108 healthy controls and 126 individuals diagnosed with schizophrenia. The control group included 33 men and 75 women aged between 20 and 59 years, with a mean age of 30.55 years. The patient group comprised 73 men and 53 women aged between 22 and 61 years, with a mean age of 44.2 years. Both groups participated in the process of photographing and measuring the lengths of the second and fourth digits to an accuracy of 0.01 mm.

Participants were recruited using convenience sampling. Healthy controls were drawn from hospital staff and postgraduate students at Xi'an Jiaotong-Liverpool University in Suzhou, China, while patients were recruited from the psychiatric department of the Fourth People's Hospital of Wuhu, China. Inclusion criteria for the control group required participants to be free of any diagnosed mental illness, physical injuries to the fingers, drug abuse, or alcohol abuse. For the patient group, inclusion criteria required a confirmed clinical diagnosis of schizophrenia by a psychiatrist based on the Diagnostic and Statistical Manual of Mental Disorders (DSM-5). Exclusion criteria for both groups included any history of finger injuries, substance abuse, alcohol dependence, or other medical conditions that could affect digit measurements.

The study protocol was approved by the Xi'an Jiaotong-Liverpool University Ethics Committee (approval number: EXT 20-01-09), and all participants provided informed consent prior to their involvement. Data collection was standardized to ensure consistency in digit measurements, and all measurements were conducted by trained personnel using calibrated equipment. This sampling and exclusion process aimed to minimize confounding factors and ensure the reliability of the findings.

Procedures

Firstly, the finger lengths were measured using photocopies of the participants' hands. To ensure consistency in the measurements, participants were instructed to stretch their palms and fingers as straight and flat as possible. They placed their left and right hands on a horizontal surface, ensuring that the entire hand, including the fingertips and creases, was clearly visible. A camera was positioned parallel to the horizontal surface to capture high-resolution images of the hands. It was emphasized that the photocopies needed to be clear and show distinct creases, as these creases served as reference points for accurate measurement.

For the photographic measurements, Adobe Photoshop (Adobe Inc., San Jose, California, United States) was used to analyze the images and measure the lengths of the index finger (2D) and ring finger (4D). The software's measurement tools allowed evaluators to precisely mark the fingertip and the crease at the base of each finger, ensuring accurate measurements. To enhance reliability, multiple evaluators independently measured the finger lengths using Photoshop, and any discrepancies were resolved through discussion or by consulting a third evaluator.

After the measurements were completed, the digit ratios (2D:4D) were calculated by dividing the length of the index finger by the length of the ring finger for both the left and right hands. This calculation was performed for each participant, and the results were recorded systematically.

The process was carefully designed to minimize variability and ensure that the procedure could be replicated by other researchers.

Statistical analysis

Data analysis was conducted with IBM SPSS Statistics for Windows, Version 26.0 (Released 2019; IBM Corp., Armonk, New York, United States), and Python 3 (Python Software Foundation, Wilmington, North Carolina, United States). The means and variances of the digit ratio (2D:4D) were computed in two groups separately for men and women. Also, the comparison of the variables between different groups was carried out by using both the independent-samples t-test and paired-samples t-test.

Then our study employed the correlation analysis to identify the main effects to the 2D:4D ratios for both the age and the gender groups (the control and the patient group) for the purpose of testing sex dimorphism. In addition, Pearson's correlation coefficients between the 2D:4D ratio and age in the two groups were applied to test the main effect. Besides, the test of computing the point-biserial correlation by SPSS to focus on the strength of the association between a continuous variable and a binary variable was effective in predicting the associations between sex (the binary variable) and the 2D:4D ratio (the continuous variable) in both hands. The significance level of the correlation analysis is 0.05.

## Results

Mean values and distributions of the 2D:4D ratio between genders

Table 1 provides a summary of the population characteristics, including the total number of participants, sex distribution, age range, and mean age with standard deviation for both the healthy control group and the schizophrenia group.

**Table 1:** Demographic characteristics of the study participants

Group	Total participants	Men (n, %)	Women (n, %)	Age range (years)	Mean age±SD (years)
Controls	108	33 (30.6%)	75 (69.4%)	20-59	30.55±8.72
Schizophrenics	126	73 (42.1%)	53 (42.1%)	22-61	44.2±9.15

The results in the control group demonstrate that the sexual dimorphism is held in the 2D:4D ratio, with the trend being more pronounced in the left hands. However, in the patient group, this common feature has not been discovered. An independent-samples t-test revealed a significant influence of sex on 2D:4D ratios of both hands. For the left hand in the control group, with an effect size (Cohen's d) of 0.40, indicating a moderate effect, the t-test results were as follows: t(106)=3.086 and p=0.003. The 95% confidence interval for the mean difference was as follows: (0.05, 0.20). For the right hand, with an effect size (Cohen's d) of 0.38, also indicating a moderate effect, the t-test results were as follows: t(106)=2.936 and p=0.004. The 95% confidence interval for the mean difference was as follows: (0.04, 0.18) (Table 2 and Table 3).

**Table 2:** The left-hand 2D:4D ratio in different groups t-test; ^*^p<0.05

Group	n	Mean (SD)			t-value	Sex difference (p, two-tailed)
		Left hand				
		2D	4D	2D:4D		
Schizophrenics						
Men	73	25.624 (2.2054)	27.0623 (1.9992)	0.9475	0.009	0.927
Women	63	25.6617 (2.5325)	27.0873 (2.6282)	0.9483	-	-
Controls						
Men	33	31.9015 (4.0637)	33.8873 (4.2999)	0.9418	3.086	0.003*
Women	75	30.1656 (4.6841)	31.3060 (5.0000)	0.965	-	-

**Table 3:** The right-hand 2D:4D ratio in different groups t-test; *p<0.05

Group	n	Mean (SD)			t-value	Sex difference (p, two-tailed)
		Right hand				
		2D	4D	2D:4D		
Schizophrenics						
Men	73	25.5764 (2.4345)	26.9855 (2.2246)	0.9479	0.0094	0.993
Women	63	25.4413 (2.6453)	26.8710 (2.7292)	0.9478	-	-
Controls						
Men	33	31.9015 (4.0637)	33.8873 (4.2999)	0.9298	2.936	0.004*
Women	75	30.1656 (4.6841)	31.3060 (5.0000)	0.9562	-	-

In contrast, the 2D:4D ratio for patients did not differ significantly between genders (left hand: t(124)=0.009 and p=0.927; right hand: t(124)=0.0094 and p=0.993). The effect sizes for these comparisons were negligible, and the confidence intervals for the mean differences included zero, suggesting no meaningful difference. In the current patient sample, the 2D:4D ratio is not sexually dimorphic.

**Figure 1:**
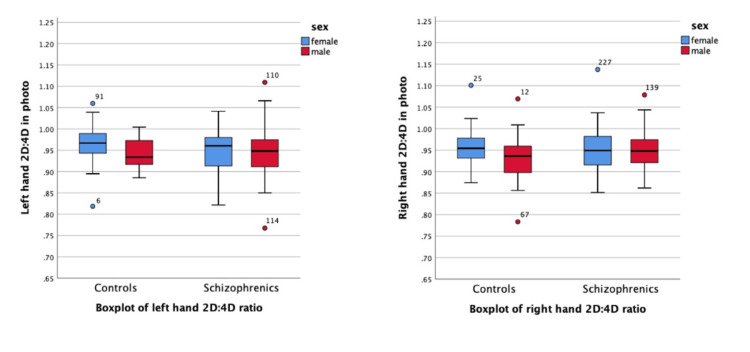
Boxplot of 2D:4D ratio across groups

Figure 1 presents the boxplot illustrating the 2D:4D ratios of both the left and right hands, stratified by sex and group (healthy controls vs. individuals with schizophrenia). When comparing 2D:4D ratios between diagnostic groups within the same sex, women in the control group exhibited slightly higher median ratios than women with schizophrenia for both hands. Among men, the median ratios were similar between groups. Notably, greater variability and more extreme outliers were observed in the schizophrenia group for both sexes.

Comparison between groups within the same gender

Table 4 and Table 5 show the mean of the 2D:4D ratio with the same gender controlled.

**Table 4:** Mean values of 2D:4D ratio in both female patients and controls t-test; *p<0.05

	Controls (N=75)	Schizophrenics (N=63)	t-value	Group difference (p, two-tailed)
	Mean (SD)	Mean (SD)		
Left-hand 2D:4D in photo	0.9650 (0.0363)	0.9483 (0.0451)	2.363	0.02*
Right-hand 2D:4D in photo	0.9562 (0.0391)	0.9478 (0.0514)	1.064	0.29

**Table 5:** Mean values of 2D:4D ratio in both male patients and controls t-test; *p<0.05

	Controls (N=33)	Schizophrenics (N=73)	t-value	Group difference (p, two-tailed)
	Mean (SD)	Mean (SD)		
Left-hand 2D:4D in photo	0.9418 (0.0352)	0.9475 (0.0552)	1.785	0.586
Right-hand 2D:4D in photo	0.9298 (0.0509)	0.9479 (0.0470)	0.546	0.077

In the right hand, the p-value was marginally above the significance level (0.05) from the result of testing the difference in mean values of 2D:4D ratio of controls and schizophrenics (t=1.785; p=0.077) for men, while in the left hand (t=0.546; p=0.586), there was essentially no difference between groups.

For women, the mean value of the 2D:4D ratio for the control group was greater than the patient group for both hands, particularly the left (left hand: t=2.363 and p=0.02; right hand: t=1.064 and p=0.29).

**Figure 2:**
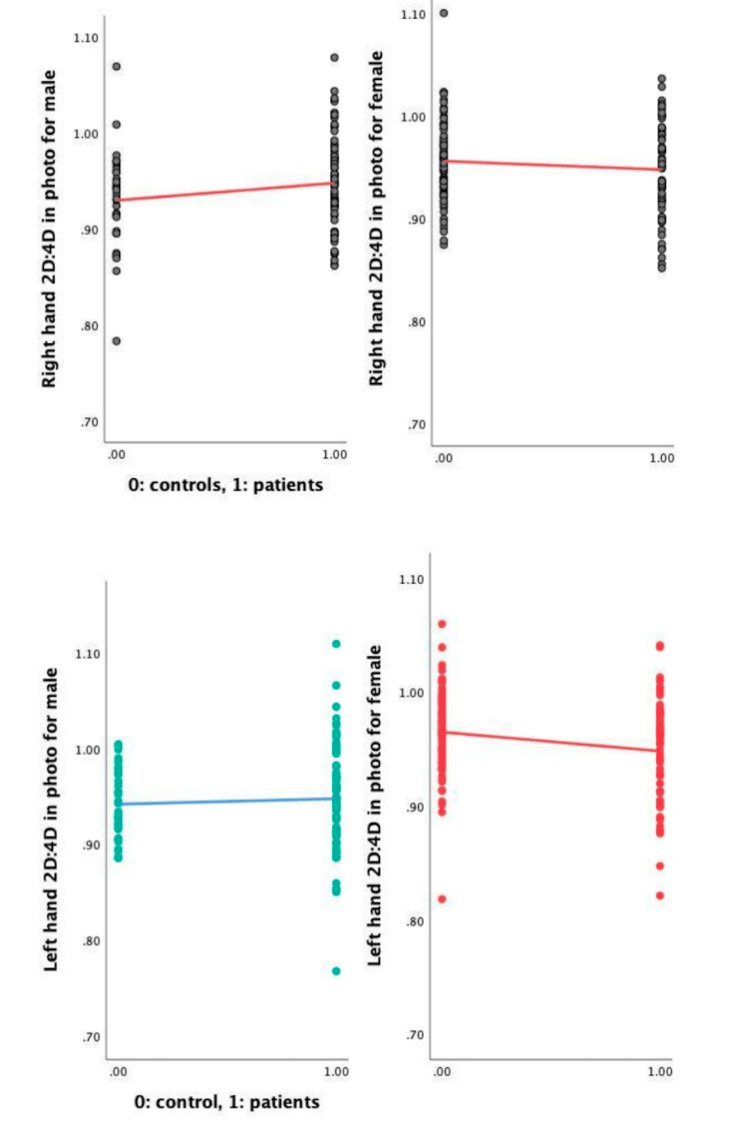
Scatter plot of 2D:4D ratio across groups

Despite the fact that the digit ratios in the two male groups were not substantially different, according to the scatter plot (Figure 2), male schizophrenics reflected a less masculine pattern in both the left and right hands, compared with the control group, while the female patients did not contribute to that trend. The women reflected a more masculine pattern, compared with the control group.

Correlation and the 2D:4D ratio between genders

In both groups, Table 6 showed the point-biserial correlation between gender and the left-hand and right-hand 2D:4D ratio.

**Table 6:** Correlation between 2D:4D ratio and sex in different groups **p<0.01

		Control group		Patient group	
		Left-hand 2D:4D ratio	Right-hand 2D:4D ratio	Left-hand 2D:4D ratio	Right-hand 2D:4D ratio
Sex	Point-biserial correlation	-0.287^**^	-0.274**	0.008	-0.001
	Sig. (two-tailed)	0.003	0.004	0.927	0.993
	N	108	108	136	136

There was a significant correlation between the gender in the control group (left hand: r=-0.287 and p=0.003<0.01; right hand: r=-0.274 and p=0.004<0.01). There are no connections between the gender and left hand (r=0.008; p=0.927) or right hand (r=-0.001; p=0.993) in the patient group.

These findings show that gender has a correlation with the 2D:4D ratio in the control group alone, corroborating the earlier conclusion that the 2D:4D ratio is associated with sexual dimorphism in healthy adults compared to patients.

Correlation and the 2D:4D ratio between ages

Table 7 and Table 8 indicate the correlations with associated p-values between current age and 2D:4D ratio in both controls and schizophrenia patients. Apart from a marginally positive correlation trend between age and left-hand 2D:4D ratio in male schizophrenics (r=0.288; p=0.047), there were no significant correlations between age and 2D:4D ratio in both groups.

**Table 7:** Correlation between age and left-hand 2D:4D ratio ^*^p<0.05

		Control group			Patient group		
		Total	Men	Women	Total	Men	Women
Age	Pearson's correlation	-0.152	-0.17	-0.127	0.212	0.288*	0.055
	Sig. (two-tailed)	0.117	0.925	0.278	0.064	0.047	0.778
	N	108	33	75	77	48	29

**Table 8:** Correlation between age and right-hand 2D:4D ratio ^*^p<0.05

		Control group			Patient group		
		Total	Men	Women	Total	Men	Women
Age	Pearson's correlation	-0.063	-0.135	0.136	0.079	0.103	0.018
	Sig. (two-tailed)	0.518	0.455	0.246	0.493	0.486	0.926
	N	108	33	75	77	48	29

## Discussion

The existence of sexual dimorphism in the 2D:4D ratio was confirmed by our research. In the control group, the digit ratio was much higher for women than for men, particularly in the left hand. The female control group's digit ratios were obviously higher than the patient group's. Furthermore, because of the apparent rise in the digit ratio for male patients, the less manly pattern has been noted more in the right hand.

The differences in the 2D:4D ratio of women and men may be induced by sex hormones when comparing the 2D:4D ratio between genders (prenatal testosterone and prenatal estrogen). Furthermore, a high ratio denotes a high level of prenatal estrogen exposure compared to prenatal testosterone, whereas a low ratio denotes the opposite trend [12,13]. According to Brown et al.'s study [8], testosterone secretion has an effect on the development of the finger skeleton in humans, which could lead to a decreased male-to-female ratio in the control group.

The less masculine pattern could be attributable to changes in the level of the prenatal sex hormone, which could be seen in the men's trend in both the control and treatment groups. Furthermore, because Hox genes are important for skeleton formation, disturbances in prenatal sex hormones may influence the control of the Hox genes' expression [8]. For instance, according to Procopio et al., the ratio might be deemed typical of fetal sex hormone concentrations indirectly based on particular studies about the proportions between the second digit length and female height [17]. As a result, the different patient group outcomes in our study would reflect aberrant prenatal estrogen or prenatal testosterone in the fetal era. Similarly, the findings are to some extent compatible with the investigations of Qian et al. stated above, particularly the much higher digit ratio for male patients [19]. However, our findings, on the other hand, show substantial variations in the 2D:4D ratio of women between the control and patient groups. Considering digit ratio as a biomarker of prenatal sex hormone secretion, there might be a link between schizophrenia and prenatal sex hormones.

Prior research revealed a higher 2D:4D ratio of the right hand than the left hand between genders. Nevertheless, our study showed that the difference was more significant in the left-hand ratio between the two groups, which is consistent with the research of Geetha et al. and the study in the population in North India [14,15].

However, the question of whether the 2D:4D ratio remains entirely invariant throughout life or is influenced by age-related factors has been the subject of ongoing debate in the literature. The purpose of investigating the correlation between age and the 2D:4D ratio in the controls and the patients is to identify whether the 2D:4D ratio and sexual dimorphism would be changing along with age. In our study, we observed a weakly significant correlation between the left-hand 2D:4D ratio and age in men within the patient group. While this finding was not robust, it suggests that circulating postnatal sex hormones might play a role in influencing the digit ratio later in life. Testosterone and estrogen levels fluctuate throughout an individual's lifespan, particularly during puberty and aging, and these hormonal changes could potentially impact the 2D:4D ratio in subtle ways. However, the mechanisms underlying such changes remain speculative and require further investigation.

This study offers several strengths. By directly examining the 2D:4D ratio, a well-recognized retrospective biomarker of prenatal sex hormone exposure, across both healthy controls and individuals with schizophrenia, the research provides empirical support for the neurodevelopmental hypothesis of schizophrenia. The application of rigorous statistical methods, including independent-samples and paired-samples t-tests, enabled an analysis of group-specific and sex-specific digit ratio patterns. The presence of statistically significant sexual dimorphism in the control group, contrasted with its absence in the schizophrenia group, offers valuable insight into potential hormonal disruptions associated with the disorder. Conducting the study in a naturalistic clinical setting in eastern China also contributes to the cultural and ecological diversity of the literature.

Nonetheless, several limitations should be acknowledged. First, the relatively small sample size of the control group, particularly the limited number of male participants (33 men vs. 75 women), may reduce the generalizability of the findings and introduce gender imbalance. This inhomogeneity may have influenced the observed digit ratio patterns, particularly the more masculinized ratios found in female patients. Second, the study did not control for potential biological and medical confounders, such as maternal health conditions, congenital abnormalities, or chronic diseases that could influence 2D:4D ratios. For instance, individuals with CAH have been shown to exhibit lower 2D:4D ratios due to elevated prenatal androgen levels [13]. The potential inclusion of such cases may introduce bias and affect the robustness of our conclusions. Another limitation of this study is that finger lengths were measured using photographs rather than advanced imaging techniques. Although this method is widely accepted and practical, it may lack the anatomical precision of methods such as CT or MRI. Future research should consider incorporating imaging-based approaches to improve measurement accuracy and reproducibility. Additionally, one limitation of this study is the lack of population diversity, as all participants were of Chinese ethnicity. This homogeneity may limit the generalizability of the findings to other racial or ethnic groups, given that 2D:4D ratios may vary across populations. Future studies should aim to include more diverse samples to improve the external validity of the results.

Future research should aim to address these limitations by recruiting larger and more gender-balanced samples, by incorporating detailed medical histories to control for relevant biological confounders, and by applying more accurate approaches. In addition, longitudinal study designs would allow for the exploration of causal relationships between prenatal hormone exposure and the onset of schizophrenia. Integrating 2D:4D ratio analysis with neuroimaging, hormonal assays, and genetic profiling may provide a more comprehensive understanding of the disorder's biological underpinnings. If validated in larger and independent cohorts, the digit ratio may potentially serve as a useful component in early screening, risk stratification, or individualized treatment planning for schizophrenia.

While the 2D:4D ratio is not a standalone diagnostic tool, it could serve as a supplementary biomarker for identifying individuals at higher risk for schizophrenia. The altered sexual dimorphism in digit ratios may provide additional evidence of neurodevelopmental abnormalities associated with the disorder. When combined with other biomarkers, such as neuroimaging findings or genetic data, the digit ratio could enhance early detection efforts, particularly in individuals with a family history of schizophrenia or other risk factors. Understanding the role of prenatal hormone exposure in schizophrenia could inform personalized treatment approaches. For example, patients with evidence of disrupted sexual dimorphism in digit ratios may benefit from interventions targeting hormone-related pathways or neurodevelopmental processes. Hormonal therapies, such as estrogen modulation, have already shown promise in improving cognitive and symptomatic outcomes in schizophrenia, particularly in female patients. Further research into the relationship between digit ratios, prenatal hormone exposure, and treatment response could help refine these approaches.

## Conclusions

Our study employed multiple statistical methods to examine the association between the 2D:4D ratio and schizophrenia, utilizing digit ratio as a retrospective marker of prenatal sex hormone exposure. We found that sexual dimorphism in 2D:4D ratios was evident among healthy individuals but absent in patients with schizophrenia. Notably, female patients exhibited a more masculinized digit ratio pattern compared to healthy women, particularly in the left hand. No significant associations were observed between age and digit ratio in either group.

These findings suggest a potential disruption in sex hormone-related neurodevelopmental pathways in schizophrenia. While digit ratio alone may not serve as a definitive diagnostic marker, it could contribute to a broader set of screening or predictive tools in the future, particularly in combination with other biological and clinical indicators. Further research is needed to explore the mechanistic links between digit ratio, prenatal and postnatal sex hormones, and the pathophysiology of schizophrenia.
